# Knockdown of circ-Gatad1 alleviates LPS induced HK2 cell injury via targeting miR-22-3p/TRPM7 axis in septic acute kidney

**DOI:** 10.1186/s12882-024-03513-1

**Published:** 2024-03-05

**Authors:** Pan Zhang, Enwei Guo, Limin Xu, Zhenhua Shen, Na Jiang, Xinghui Liu

**Affiliations:** 1 Department of Clinical Laboratory, Gongli Hospital of Shanghai Pudong New Area, 219 Miao Pu Road, 200135 Shanghai, China; 2 Department of Intensive Care Unit, Gongli Hospital of Shanghai Pudong New Area, 219 Miao Pu Road, 200135 Shanghai, China

**Keywords:** circ-Gatad1, HK2, miR-22-3p, TRPM7, Septic acute kidney

## Abstract

**Background:**

Sepsis is a life-threatening, systemic inflammatory disease that can lead to a variety of conditions, including septic acute kidney injury (AKI). Recently, multiple circular Rnas (circRNAs) have been implicated in the development of this disease.

**Methods:**

In this study, we aimed to elucidate the role of circ-Gatad1 in sepsis induced AKI and its potential mechanism of action. High-throughput sequencing was used to investigate abnormal expression of circRNA in AKI and healthy volunteer. Bioinformatics analysis and luciferase reporting analysis were used to clarify the interacted relationship among circRNA, miRNA and mRNA. HK2 cells were treated with lipopolysaccharide (LPS) to establish septic AKI cell model. HK2 cells were employ to analysis the ROS, inflammatory cytokines expression, proliferation and apoptosis under LPS condition.

**Results:**

The result show that the expression of circ-Gatad1 was increased in septic acute kidney patients. Downregulation circ-Gatad1 suppressed LPS-treated induced HK2 cells injury including apoptosis, proliferation ability, ROS and inflammatory cytokines level. Bioinformatics and luciferase report analysis confirmed that both miR-22-3p and TRPM7 were downstream targets of circ-Gatad1. Overexpression of TRPM7 or downregulation of miR-22-3p reversed the protective effect of si-circ-Gatad1 to HK2 after exposure to LPS (5 µg/ml) microenvironment.

**Conclusion:**

In conclusion, knockdown of circ-Gatad1 alleviates LPS induced HK2 cell injury via targeting miR-22-3p/TRPM7 axis in septic acute kidney.

## Background

Septic shock with acute kidney injury (AKI) is common in critically ill patients [1]. Although the in-hospital mortality rate for patients receiving kidney replacement therapy in ICU decreased from 44.9% in 2007 to 36.1% in 2016, the mortality rate for patients with sepsis remained high, at > 50% [2]. AKI is a critical and severe clinical disease caused by a variety of factors. Lipopolysaccharide (LPS)-induced sepsis is a primary cause of AKI in critically ill patients [3, 4]. Therefore, LPS is always utilized to construct septic AKI cell models in renal cells in vitro [5]]. Recent studies have confirmed that the pathogenesis of sepsis induced AKI includes a series of complex interactions such as vascular endothelial cell dysfunction, inflammation and renal tubule cell apoptosis [6, 7]. However, efforts to translate these findings from the laboratory to clinical trials have proved unsuccessful. Therefore, it is necessary to fully understand the pathogenesis of septic kidney injury in order to develop more effective treatment strategies.

Circular ribonucleic acid (circRNA) is a widely available non-coding RNA with a closed-loop structure and high stability [8, 9]. There is growing evidence that many circrnas are strongly associated with a variety of human diseases, including sepsis [10, 11]. Previous studies have confirmed that circ-UBE2D2 mitigates the progression of SAKI in rats by targeting miR-370-3p/NR4A3 axis [12]. Circ-RASGEF1B promotes LPS-induced apoptosis and inflammatory response by targeting miRNA-146a-5p/PDK1 axis in septic acute kidney injury cell model [13]. Knockdown of circ-FANCA alleviates LPS-induced HK2 cell injury via targeting miR-93-5p/OXSR1 axis in septic acute kidney injury [11]. However, the function of circRNA in the progression of sepsis-induced AKI and the underlying mechanisms involved remain largely unknown.

In the current study, we found that the expression of circ-Gatad1 was increased in septic acute kidney. We constructed septic AKI model in HK2 cells with the stimulation of LPS. The expression pattern of circ-Gatad1 and its function were investigated in LPS-treated HK2 cells. Meanwhile, circ-Gatad1 was found to act a sponge for miR-22-3p. Mechanistically, the regulatory function of circ-Gatad1/miR-22-3p/mRNA network was explored, which might provide a new perspective for the pathogenesis of sepsis-related AKI.

## Materials and methods

### Serum samples

The serum specimens were collected from 20 fasting septic AKI patients and 20 fasting healthy subjects at Shanghai Pudong New Area Gongli Hospital. These serum specimens were acquired by centrifugation and then preserved at − 80 °C prior to use. Written informed consent from all participants. The clinicopathologic characteristics of sepsis-AKI patients was listed in Table 1.

**Table 1:** Clinicopathologic features of Sepsis-AKI patients

Parameters	Normal group	Sepsis-AKI group
Number of patients	20	20
Gender (male/female)	11/9	12/8
Age (years)	53.2 ± 5.2	55.6 ± 4.6
Serum creatinine (µmol/L)	64.5 ± 5.7	322.8 ± 31.5
Blood urea nitrogen (mmol/L)	4.4 ± 0.5	22.6 ± 1.2
CRP (mg/L)		182 ± 18.8
Calcitonin original (ng/L)		22.6 ± 2.8

### Strand-specific high-throughput RNA-Seq library construction

Total RNA from serum of AKI patients were extracted with TRIzol reagent (Invitrogen, Carlsbad, CA, USA). Then 3 µg total RNA were used remove ribosomal RNA, and retain RNA classes including noncoding RNAs with VAHTS Total RNA-seq (H/M/R) Library Prep kits from Illumina (Vazyme Biotech Co., Ltd, Nanjing, China). We treated RNA through 40 U RNase R (Epicenter) at 37 °C for three hours, followed by TRIzol purification. We prepared RNA-seq libraries via KAPA Stranded RNA-Seq Library Prep kits (Roche, Basel, Switzerland) and used them for deep sequencing (Illumina HiSeq 4000 at Aksomics, Inc., Shanghai, China).

### Cell culture, LPS induction and cell transfection

Human renal tubule epithelial cells (Human kidney-2, HK2) were purchased from Procell (Wuhan, China) and cultured in Minimum Essential Medium (MEM; Procell) plus 10% fetal bovine serum (FBS; Procell) and 1% penicillin–streptomycin (Procell) in a 37 °C, 5% CO_2_ humid incubator. HK2 cells were treated with LPS at different concentrations (0–15 µg/mL) for 24 h. For the establishment of sepsis-related AKI model in vitro, HK2 cells in the following experiments were exposed to 5 µg/mL LPS (Solarbio, Beijing, China) for 12 h, while the control HK2 cells were cultivated with MEM medium simultaneously.

TRPM7 overexpression vector were constructed by insert cDNA of TRPM7 into the pcDNA3.1 vector. miR-22-3p inhibitor and siRNA against circ-Gatad1 (si-circ-Gatad11) were synthesized by Genepharma (Suzhou, China). Cell transfection was then performed using Lipofectamine 2000 (Invitrogen) according to the manufacturer’s instructions.

### Cell counting kit‑8 (CCK-8) assay

HK2 cells with various treatments were seeded into 96-well plates (1 × 104 cells/well) and incubated for 48 h. Subsequently, 10 µL CCK-8 reagent (5 mg/mL; Beyotime, Shanghai, China) was pipetted into each well, and cells were maintained at 37 °C for another 2 h. The absorbance of each well at 450 nm measured on a microplate reader (Thermo Fisher Scientific, Waltham, MA, USA) was utilized for determining cell viability.

### Enzyme-linked immunosorbent assay (ELISA)

The release of inflammatory cytokines interleukin-1β (IL-1β), IL-6 and tumor necrosis factor α (TNF-α) in culture medium was determined using the corresponding ELISA kits against TNF-α, IL-1β and IL-6 (Beyotime) according to the user’s manual. The colorimetric changes were determined at 450 nm.

### RNA isolation and real-time PCR

Total RNA was extracted with TRIzol Reagent (Invitrogen), followed by cDNA synthesis using a TransScript All-in-One First-Strand cDNA Synthesis SuperMix (Transgen Biotech, Beijing, China), was performed. PCR was performed using a Bio-Rad PCR instrument (Bio-Rad, Hercules, CA, U.S.A.) with 2× Taq PCR Master Mix (Solarbio, Beijing, China) following the manufacturer’s instructions. The fold changes were calculated by means of relative quantification (2^−△△Ct^ method).

### Dual-luciferase reporter assay

The 3’UTR of HK2 gene and circ-Gatad1 containing the predicated binding sites for miR-22-3p were amplified and inserted into the multiple cloning sites of pMIR-REPORT luciferase reporter vector (Ambion, Austin, U.S.A.). Then, HK2 cells were co-transfected with 0.1 µg of luciferase reporter vectors comprising wild-type or mutant type of 3’UTR of HK2 or circ-Gatad1 and either miR-22-3p mimic or miR-control by Lipofectamine 2000 (Invitrogen, Carlsbad, U.S.A.). Relative luciferase activity was calculated by normalizing the firefly luminescence to the Renilla luminescence using the Dual-Luciferase Reporter Assay System (Promega, Madison, WI, U.S.A.) according to the manufacturer’s instructions at 48 h post-transfection.

### Reactive oxygen species (ROS) activity

Intracellular generation of ROS in HK2 cells or wound skin tissue were detected using Dihydroethidium (DHE) assay (Beyotime, China). Fluorescence was measured at 488 nm excitation and 525 nm emission using a fluorescence microplate reader (PerkinElmer, USA).

### Statistical analysis

Continuous variables were denoted by means ± SD (standard deviation). We used one-way variance analysis for the comparisons by GraphPad Prism (version 5.0; GraphPad, La Jolla, USA). *P* ≤ 0.05 indicated statistical significances.

## Results

### The expression of circ-Gatad1 was increased in septic acute kidney patients

**Fig. 1:**
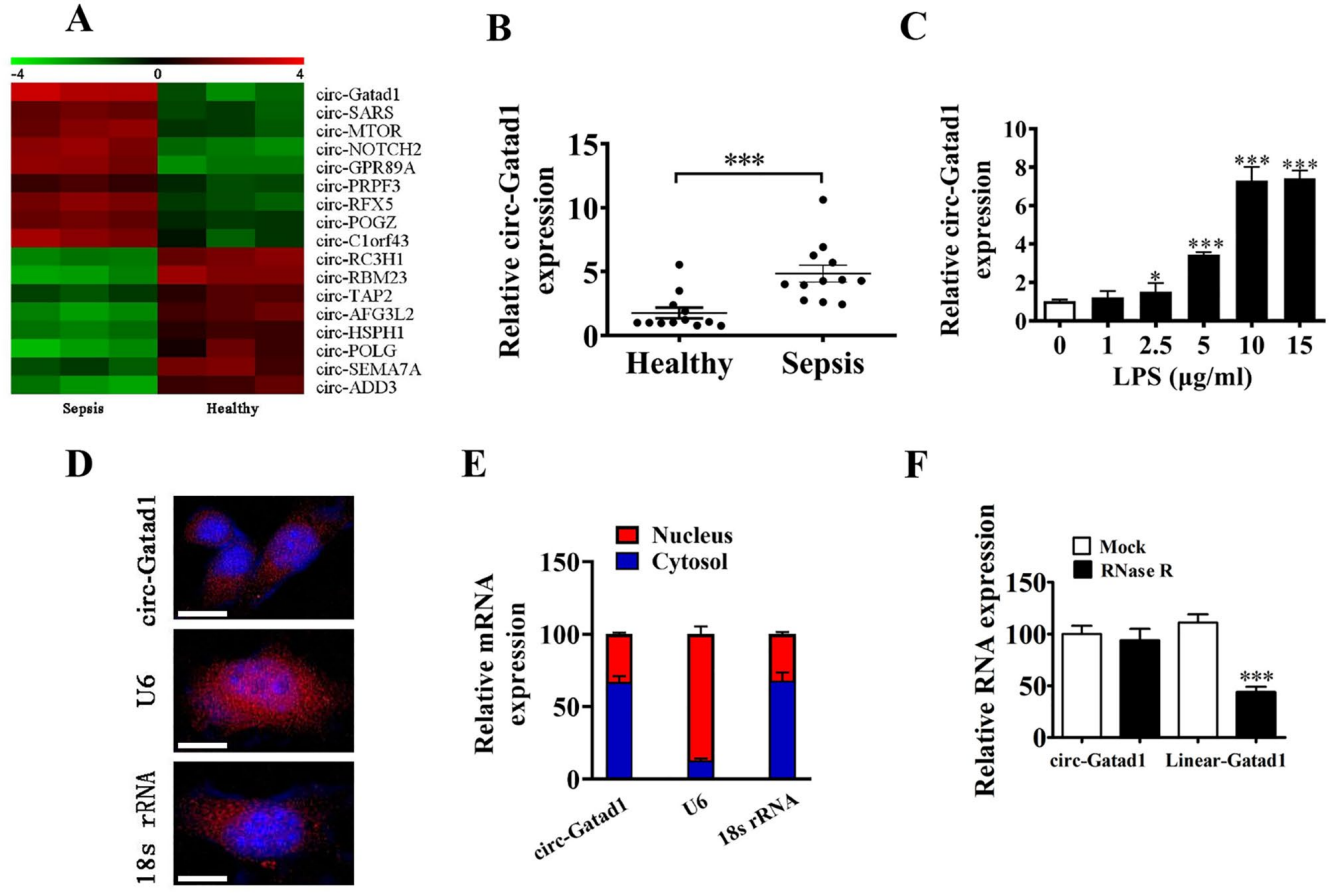
The expression of circ-Gatad1 was increased in septic acute kidney. (**A**) High-throughput sequencing analysis identified a series of upregulated and downregulated circRNAs between healthy and AKI. (**B**) RT-qPCR detection show that the expression of circ-Gatad1 was increased in septic acute kidney patients. Data are expressed as mean ± SD. ^***^p < 0.001 vs. Healthy. (**C**) RT-qPCR detection show that the expression of circ-Gatad1 was increased in HK2 cells after treatment with different dose of LPS (0–15 µg/ml). Data are expressed as mean ± SD. ^***^p < 0.001 vs. 0 µg/ml. (**D** and **E**) Representative FISH images and subcellular fractionation assays showed the subcellular distribution of circ-Gatad1 in HK2 cells. U6 was used as a nuclear control and 18 S rRNA was used as a cytoplasmic control. Scales bar, 20 μm. (F) After RNase R treatment, the levels of circ-Gatad1 and linear Gatad1 mRNA were determined using qRT-PCR. Data are expressed as mean ± SD. ^***^p < 0.001 vs. Mock

High-throughput sequencing analysis identified a series of upregulated and downregulated circRNAs between healthy and AKI patient (Fig. 1A). RT-qPCR detection found that circ-Gatad1 expression was increased in septic acute kidney patients (Fig. 1B). Next, the septic AKI model in vitro was established by treating HK2 cells with LPS. And the result show that circ-Gatad1 expression was increased with the concentration increased of LPS (Fig. 1 C). 5 µg/mL LPS was selected for subsequent experiments. Since the localization of RNAs within cells are crucial for their functional display, we further determined the subcellular localization of circ-Gatad1 in HK2 cells. FISH and subcellular fractionation assays revealed that circ-Gatad1 was predominantly located in the cytoplasm (Fig. 1D and E). Meanwhile, RNase R assay showed that the level of linear Gatad1 mRNA was significantly reduced by the digestion with RNase R, while circ-Gatad1 was resistant to RNase R, indicating circ-Gatad1 was a highly stable circular RNA (Fig. 1F). These observations indicated that circ-Gatad1 might be a functional molecule in septic AKI progression.

### Downregulation circ-Gatad1 suppressed LPS-treated induced HK2 cells injury

**Fig. 2:**
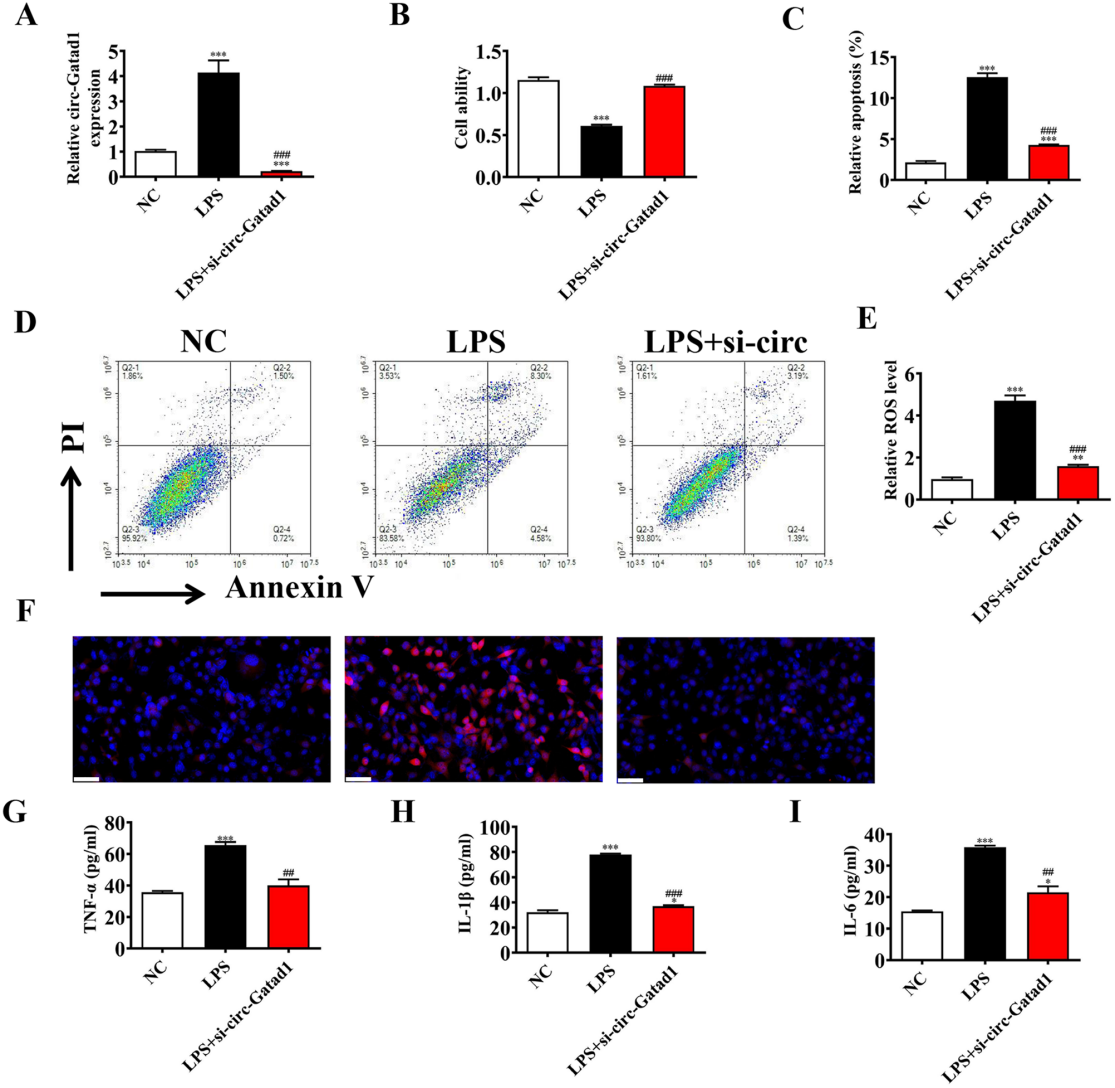
Downregulation circ-Gatad1 suppressed LPS-treated induced HK2 cells injury. (**A**) RT-qPCR detection show that the expression of circ-Gatad1 in HK2 cells after transfected with siRNA against circ-Gatad1. Data are expressed as mean ± SD. ^***^p < 0.001 vs. NC. ^###^*p* < 0.001 vs. LPS. (**B**) CCK8 detection show the cells proliferation ability in HK2 cells. Data are expressed as mean ± SD. ^***^p < 0.001 vs. NC. ^###^*p* < 0.001 vs. LPS. (**C** and **D**) Flow apoptosis detection show the apoptosis of HK2 cells. Data are expressed as mean ± SD. ^***^p < 0.001 vs. NC. ^###^*p* < 0.001 vs. LPS. (**E** and **F**) Dihydroethidium (DHE) staining show the ROS level in HK2 cells. Data are expressed as mean ± SD. ^**^p < 0.01, ^***^p < 0.001 vs. NC. ^###^*p* < 0.001 vs. LPS. Scales bar, 50 μm. (**G**-**I**) ELISA detection show the expression of inflammatory factor TNF-α, IL-1β, IL-6. Data are expressed as mean ± SD. Data are expressed as mean ± SD. ^*^p < 0.05, ^***^p < 0.001 vs. NC. ^##^*p* < 0.01, ^###^*p* < 0.001 vs. LPS

RT-qPCR detection show that circ-Gatad1 expression was decreased significantly after transfected with siRNA against circ-Gatad1 even in LPS-treated condition in HK2 cells (Fig. 2A). CCK8 detection show that downregulation circ-Gatad1 restored the proliferation ability of HK2 cells under LPS (5 µg/mL) condition (Fig. 2B). Flow cytometry for HK2 cells apoptosis detection show that downregulation circ-Gatad1 reversed LPS (5 µg/mL) induced HK2 cells apoptosis (Fig. 2 C and 2D). Immunofluorescence for ROS detection show that downregulation circ-Gatad1 reversed LPS (5 µg/mL) induced ROS level in HK2 cells (Fig. 2E F). ELISA detection show that downregulation circ-Gatad1 reversed LPS (5 µg/mL) induced inflammatory factor TNF-α, IL-1β, IL-6 expression in HK2 cells (Fig. 2G-I).

### Both mir-22-3p and TRPM7 were downstream targets of circ-Gatad1

**Fig. 3:**
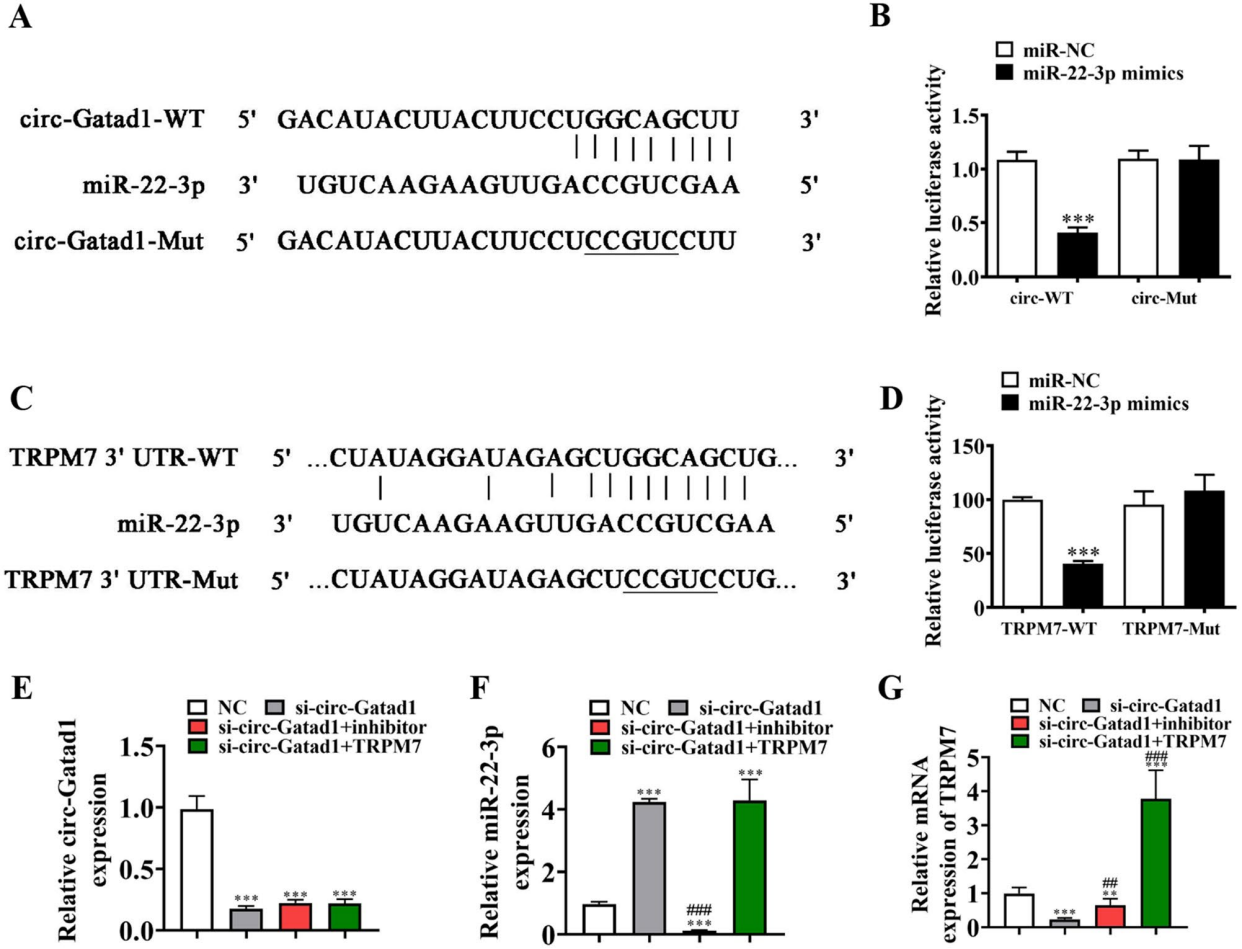
miR-22-3p and TRPM7 were downstream targets of circ-Gatad1. (**A**) Bioinformatics analysis predicting binding sites of miR-22-3p in circ-Gatad1. Mutant version of circ-Gatad1 is shown. (**B**) Relative luciferase activity determined 48 h after transfection of HK2 cells with miR-22-3p mimic/NC or circ-Gatad1 wild-type/Mut. Data are means ± SD. ***P < 0.001. (**C**) Prediction of miR-22-3p binding sites in the 3’-UTR of TRPM7. Mutant version of 3’-UTR-TRPM7 is shown. (**D**) Relative luciferase activity 48 h after transfection of HK2 cells with miR-22-3p mimic/NC or 3’-UTR-TRPM7 wild-type/Mut. Data are means ± SD. **P < 0.01. (**E-G**) RT-qPCR detection show the expression of circ-Gatad1, miR-22-3p and TRPM7 in HK2 cells. Data are expressed as mean ± SD. ^**^p < 0.01, ^***^p < 0.001 vs. NC. ^##^*p* < 0.01, ^###^*p* < 0.001 vs. si-circ-Gatad1

Bioinformatics analysis (http://starbase.sysu.edu.cn/) found that circ-Gatad1 can interacted with a series of miRNA including miR-22-3p. A luciferase reporter analysis confirmed that miR-22-3p inhibited luciferase activity in wild-type cells, but not in mutated cell lines (Fig. 3A and B), suggesting that miR-22-3p was the target of circ-Gatad1.

Bioinformatics analysis found that TRPM7 was the downstream target of miR-22-3p. To further confirm the relationship between miR-22-3p and TRPM7, wild-type or mutated 3’UTR-TRPM7 sequences containing the miR-22-3p binding sequence were constructed into a luciferase reporter vector (Fig. 3 C). The luciferase reporter vector was then transfected into HK2 cells combined with or without miR-22-3p mimic. A luciferase reporter analysis found that miR-22-3p inhibited luciferase activity in wild-type cells, but not in mutated cell lines (Fig. 2,3D), suggesting that TRPM7 was the target of miR-22-3p.

RT-qPCR results showed that circ-Gatad1 expression were decreased after transfected with siRNA against circ-Gatad1. But treatment with the miR-22-3p inhibitor or TRPM7 overexpression vector (TRPM7) have no effect to circ-Gatad1 expression in HK2 cells (Fig. 3E). Suggestion that both miR-22-3p and TRPM7 were the downstream target of circ-Gatad1. Rt-qPCR detection also found that circ-Gatad1 downregulation promotion miR-22-3p expression. TRPM7 silence can not restore miR-22-3p expression after circ-Gatad1 silence (Fig. 3F). Suggestion that miR-22-3p was at the downstream of TRPM7. The result also found that circ-Gatad1 silence decreased TRPM7 expression. But upregulation TRPM7 reversed the inhibit effect of si-circ-Gatad1 to TRPM7 expression. After transfected with TRPM7 overexpression vector, the expression of TRPM7 was significantly increased (Fig. 3G). Suggestion that circ-Gatad1 promotion TRPM7 expression by sponging miR-22-3p.

### Overexpression of TRPM7 or downregulation of mir-22-3p reversed the protective effect of si-circ-Gatad1 to HK2 after exposure to LPS (5 µg/ml) microenvironment

**Fig. 4:**
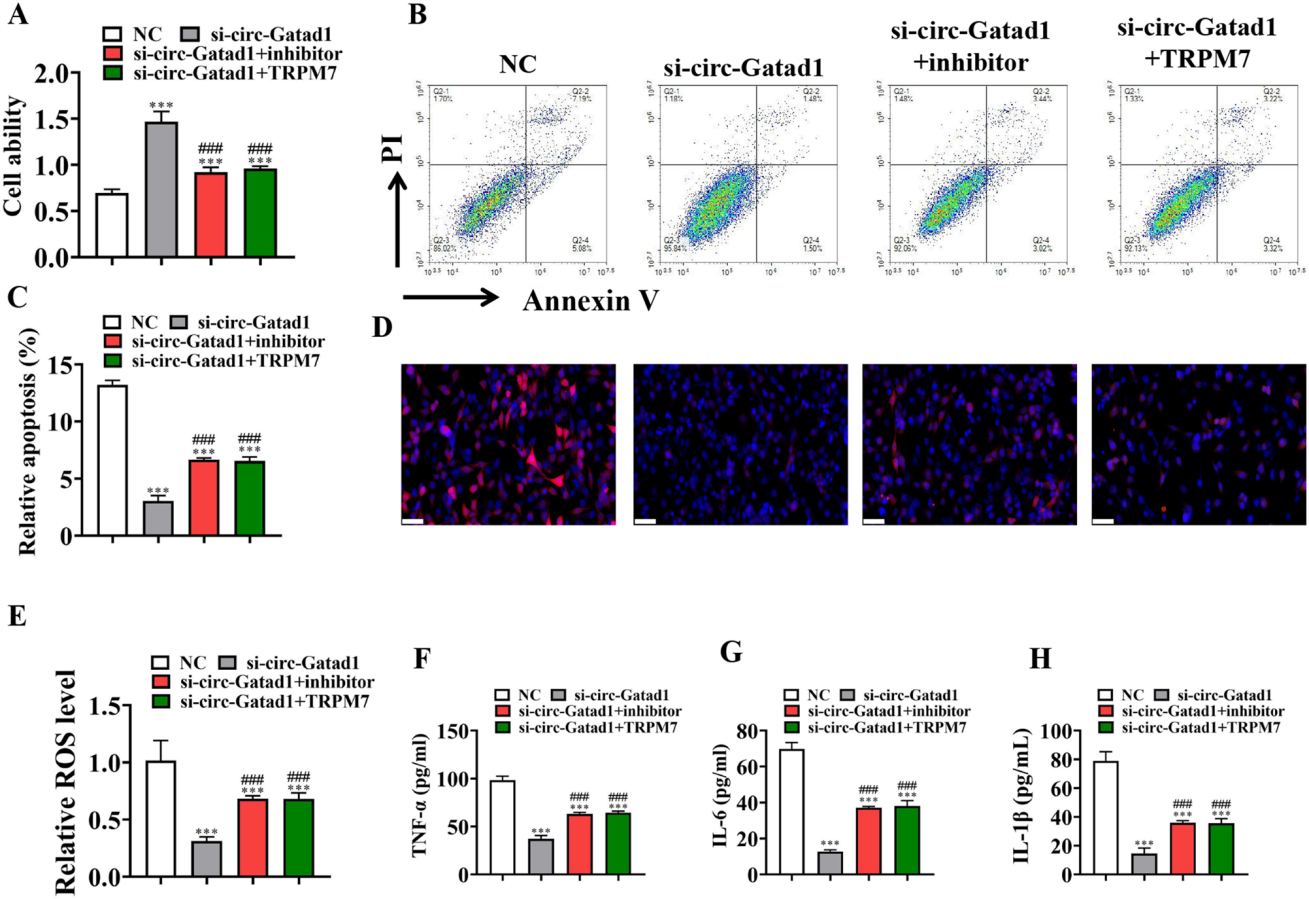
Overexpression of TRPM7 or downregulation of miR-22-3p reversed the protective effect of si-circ-Gatad1 to HK2 after exposure to LPS (5 µg/ml) microenvironment. (**A**) CCK8 detection show the cells proliferation ability in HK2 cells. Data are expressed as mean ± SD. ^***^p < 0.001 vs. NC. ^###^*p* < 0.001 vs. si-circ-Gatad1. (**B** and **C**) Flow apoptosis detection show the apoptosis of HK2 cells. Data are expressed as mean ± SD. ^***^p < 0.001 vs. NC. ^###^*p* < 0.001 vs. si-circ-Gatad1. (**D** and **E**) Dihydroethidium (DHE) staining show the ROS level in HK2 cells. Data are expressed as mean ± SD. ^***^p < 0.001 vs. NC. ^###^*p* < 0.001 vs. si-circ-Gatad1. Scales bar, 50 μm. (**F-H**) ELISA detection show the expression of inflammatory factor TNF-α, IL-1β, IL-6. Data are expressed as mean ± SD. Data are expressed as mean ± SD. ^***^p < 0.001 vs. NC. ^###^*p* < 0.001 vs. si-circ-Gatad1

CCK8 detection show that overexpression of TRPM7 or downregulation of miR-22-3p reversed the protective effect of si-circ-Gatad1 to HK2 cells proliferation under LPS (5 µg/mL) condition (Fig. 4A). Flow cytometry for HK2 cells apoptosis detection show that overexpression of TRPM7 or downregulation of miR-22-3p reversed the inhibit effect of si-circ-Gatad1 to HK2 cells apoptosis under LPS (5 µg/mL) condition (Fig. 4B C). Immunofluorescence for ROS detection show that overexpression of TRPM7 or downregulation of miR-22-3p reversed the inhibit effect of si-circ-Gatad1 to LPS (5 µg/mL) induced ROS level in HK2 cells (Fig. 4D and E). ELISA detection show that overexpression of TRPM7 or downregulation of miR-22-3p reversed the inhibit effect of si-circ-Gatad1 to LPS (5 µg/mL) induced inflammatory factor TNF-α, IL-1β, IL-6 expression in HK2 cells (Fig. 4F-H).

## Discussion

Sepsis is a major cause of acute kidney injury (AKI) among patients in the intensive care unit [14]. There is substantial evidence that patients diagnosed with septic AKI have a higher risk of death than those with non-septic AKI and generally have longer ICU and hospital stays [15–17]. Therefore, it is of great significance to identify the specific pathogenesis for finding new therapies for septicemic AKI. Therefore, it is of great significance to identify the specific pathogenesis for finding new therapies for septicemic AKI. In this study, we found that circRNA play an important role in regulation the progress of AKI. High-throughput sequencing analysis found that circ-Gatad1 expression was increased. In vitro simulation experiment show that circ-Gatad1 expression was increased with LPS induction and in dose dependent way. Similar to other circRNAs, circ-Gatad1 is primarily located in the cytoplasm [18, 19].

The study also found that downregulation circ-Gatad1 reversed LPS induced proliferation inhibition. And circ-Gatad1 silence also inhibit LPS induced HK2 cells apoptosis, ROS accumulation and inflammatory factor expression. Sepsis caused by lipopolysaccharide (LPS) endotoxin is a common cause and trigger of AKI. Sepsis caused by lipopolysaccharide (LPS) endotoxin is a common cause of AKI and a trigger for inflammatory response [20]. Its pathogenesis includes renal endothelial cell injury, renal cell apoptosis, overproduction of multiple inflammatory mediators, superoxide injury and inflammatory immune response [21–23]. Its pathogenesis includes renal endothelial cell injury, renal cell apoptosis, excessive production of multiple inflammatory mediators, superoxide injury [24].

The study also found that miR-22-3p and transient receptor potential melastatin 7 (TRPM7) were downstream targets of circ-Gatad1. Luciferase report analysis confirmed that circ-Gatad1 can interacted with miR-22-3p. Downregulation circ-Gatad1 promotion miR-22-3p expression. Previous studies have confirmed that miR-22-3p was significantly down-regulated in sepsis-induced acute kidney injury, in vivo and LPS-induced sepsis model in HK-2 cells [25]. miR-22 alleviates sepsis-induced acute kidney injury via targeting the HMGB1/TLR4/NF-κB signaling pathway [26]. Deregulated miR-22-3p in patients with sepsis-induced acute kidney injury serves as a new biomarker to predict disease occurrence and 28-day survival outcomes [27]. In this study, we also found that inhibit miR-22-3p reversed the protective effect of si-circ-Gatad1 to HK2 after exposure to LPS (5 µg/ml) microenvironment. Suggestion that circ-Gatad1 regulation the progress of AKI by sponging miR-22-3p.

Further study found that miR-22-3p can interacted with 3’UTR of TRPM7 which was confirmed with luciferase report analysis. Previous studies have confirmed that TRPM7 was upregulated in LPS-treated cells, and knocking improved cell viability, reduced cells apoptosis, inflammation, and oxidative stress [28]. The study also found that TRPM7 in renal ischemia-reperfusion injury might provide new mechanistic insights for a potential biomarker as diagnostic and therapeutic target of AKI [29]. Our study found that overexpression TRPM7 reversed the protective effect of si-circ-Gatad1 to HK2 after exposure to LPS (5 µg/ml) microenvironment. Suggestion that circ-Gatad1 regulation the progress of AKI by sponging miR-22-3p and promotion TRPM7 expression.

## Conclusion

Taken together, our results show that circ-Gatad1 promoted LPS-induced HK2 cell injury via regulating the miR-22-3p/TRPM7 signaling pathway, suggesting that circ-Gatad1 knockdown might be a potential pathway for alleviating sepsis-induced AKI.

## Data Availability

The datasets used and/or analyzed during the current study are available from the corresponding author on reasonable request.
