# Correction: Knockdown of circ-Gatad1 alleviates LPS induced HK2 cell injury via targeting miR-22- 3p/TRPM7 axis in septic acute kidney

**DOI:** 10.1186/s12882-024-03580-4

**Published:** 2024-04-25

**Authors:** Pan Zhang, Enwei Guo, Limin Xu, Zhenhua Shen, Na Jiang, Xinghui Liu

**Affiliations:** 1 Department of Clinical Laboratory, Gongli Hospital of Shanghai Pudong New Area, 219 Miao Pu Road, Shanghai, 200135 China; 2 Department of Intensive Care Unit, Gongli Hospital of Shanghai Pudong New Area, 219 Miao Pu Road, Shanghai, 200135 China

**Correction: BMC Nephrology (2024) 25:79**

10.1186/s12882-024-03513-1

Following publication of the original article [1], we have been informed that the name of author Xinghui Liu has a spelling. Also, the corresponding author e-mail has been changed.

The incorrect name is: Xinhui Liu.

The correct name is: Xinghui Liu.

The original article has been corrected.

## References

[1] Zhang, et al. BMC Nephrol. 2024;2579. 10.1186/s12882-024-03513-1.

